# Complete Genome Sequence of *Curtobacterium* sp. Strain MR_MD2014, Isolated from Topsoil in Woods Hole, Massachusetts

**DOI:** 10.1128/genomeA.01504-15

**Published:** 2015-12-31

**Authors:** Richard M. Mariita, Srijak Bhatnagar, Kurt Hanselmann, Mohammad J. Hossain, Jonas Korlach, Matthew Boitano, Richard J. Roberts, Mark R. Liles, Anthony G. Moss, Jared R. Leadbetter, Dianne K. Newman, Scott C. Dawson

**Affiliations:** a Department of Biological Sciences, Auburn University, Alabama, USA; b Microbiology Graduate Group, University of California Davis, Davis, California, USA; c Department of Earth Sciences, ETH Zurich, Zürich, Switzerland; d Pacific Biosciences, Menlo Park, California, USA; e New England BioLabs, Ipswich, Massachusetts, USA; f Linde Center for Global Environmental Science, California Institute of Technology, Pasadena, California, USA; g Division of Biology and Biological Engineering, California Institute of Technology, Pasadena, California, USA; h Howard Hughes Medical Institute, Pasadena, California, USA; i Division of Geological and Planetary Sciences, California Institute of Technology, Pasadena, California, USA; j Department of Microbiology and Molecular Genetics, University of California Davis, Davis, California, USA

## Abstract

Here, we present the 3,443,800-bp complete genome sequence of *Curtobacterium* sp. strain MR_MD2014 (phylum *Actinobacteria*). This strain was isolated from soil in Woods Hole, MA, as part of the 2014 Microbial Diversity Summer Program at the Marine Biological Laboratory in Woods Hole, MA.

## GENOME ANNOUNCEMENT

Members of the genus *Curtobacterium*, first defined in 1972 by Yamada and Komagata (1), belong to the family *Microbacteriaceae*, within the phylum *Actinobacteria*. Several *Curtobacterium* isolates associate with plants as either pathogens or nonpathogens. Their cells are generally Gram-positive irregularly shaped motile rods. Here, *Curtobacterium* sp. strain MR_MD2014 was cocultured with *Streptomyces* sp. strain CCM_MD2014 from the topsoil collected near a rusted fire hydrant in Woods Hole, MA (41°31′44.65″N 70°40′21.5″W) on 7 July 2014, on starch-arginine-tryptophan (SAT) medium using protocols modified from El-Nakeeb and Lechevalier (2). This strain was then isolated from the coculture by selective culture, using antibiotic discs on a modified LB agar. The identity of each of the isolates was verified using the 16S rRNA gene sequence. This strain was cultivated as part of a student-led microbial isolation and sequencing initiative at the 2014 Microbial Diversity Summer Program at the Marine Biological Laboratory in Woods Hole, MA.

From the original coculture, total DNA extraction was carried out by 1 h of lysozyme digestion, followed by use of the Promega Wizard genomic DNA purification kit. The isolated DNA was quantified using the Promega QuantiFluor double-stranded DNA (dsDNA) system and then size selected for a minimum length of 4 kb. The size-selected DNA was sequenced on the Pacific Biosciences RSII sequencing platform with P5C3 chemistry. The sequenced fragments were assembled using HGAP3 on the SMRT Portal (3). The final assembled circular genome had a size of 3,443,800 bp, with 89× sequencing coverage and a G+C composition of 71.95%.

The genome was annotated using NCBI Prokaryotic Genome Annotation Pipeline version 2.8 (rev. 449627) (4, 5). The identified genes were composed of 2,675 coding sequences (CDSs), 4 rRNA operons, 48 tRNAs, and 1 noncoding RNA (ncRNA) gene. CRISPRFinder (6) identified one definite clustered regularly interspaced short palindromic repeat (CRISPR) and 13 potential CRISPRs in the genome. The PHAST server (7) predicted three prophages, two of which were incomplete, and one of which was of questionable quality. REBASE (8) identified 3 candidate methylase genes and one methylated motif, GG^m6^AGGC, which was found using Pacific Biosciences SMRT Portal analysis. This was unambiguously assigned to the type IIG restriction enzyme Csp2014I (see organism number 14032 on the REBASE website for details).

Phylogenetic analysis of the 16S rRNA gene sequence using SSU-align (version 0.1) (9) and RAxML (version 8.2.3) (10) revealed that strain MR_MD2014 belongs to the genus *Curtobacterium*, in the family *Microbacteriaceae*. The 16S rRNA gene-based analysis did not provide sufficient data for a refined classification of this strain at the species level. A phylogenetic reconstruction based upon 42 conserved single-copy-marker genes using CheckM (11) identified *Curtobacterium* sp. YR515 as being the most similar to the strain MR_MD2014 (see http://dx.doi.org/10.6084/m9.figshare.1574022). An average nucleotide identity (ANI) calculation using IMG (12) confirmed that the closest available genome to this strain is *Curtobacterium* sp. YR515, with only 85.3% average nucleotide identity across shared genes.

### Nucleotide sequence accession numbers.

The complete nucleotide sequence of this genome is available through GenBank under the accession no. CP009755. The version described here is CP009755.1.
